# IGF-1, Inflammation and Retinal Degeneration: A Close Network

**DOI:** 10.3389/fnagi.2018.00203

**Published:** 2018-07-05

**Authors:** Ana I. Arroba, Antonio Campos-Caro, Manuel Aguilar-Diosdado, Ángela M. Valverde

**Affiliations:** ^1^Alberto Sols Biomedical Research Institute (IIBm) (CSIC/UAM), Madrid, Spain; ^2^Spanish Biomedical Research Centre in Diabetes and Associated Metabolic Disorders (CIBERdem), ISCIII, Madrid, Spain; ^3^Research Unit, Instituto de Investigación e Innovación en Ciencias Biomédicas de la Provincia de Cádiz (INiBICA), University Hospital “Puerta del Mar”, Cádiz, Spain; ^4^Department of Endocrinology and Metabolism, Instituto de Investigación e Innovación en Ciencias Biomédicas de la Provincia de Cádiz (INiBICA), University Hospital “Puerta del Mar”, Cádiz, Spain

**Keywords:** retina, inflammation, neurodegeneration, IGF-1, microglia and exosomes

## Abstract

Retinal degenerative diseases are a group of heterogeneous diseases that include age-related macular degeneration (AMD), retinitis pigmentosa (RP), and diabetic retinopathy (DR). The progressive degeneration of the retinal neurons results in a severe deterioration of the visual function. Neuroinflammation is an early hallmark of many neurodegenerative disorders of the retina including AMD, RP and DR. Microglial cells, key components of the retinal immune defense system, are activated in retinal degenerative diseases. In the microglia the interplay between the proinflammatory/classically activated or antiinflammatory/alternatively activated phenotypes is a complex dynamic process that occurs during the course of disease due to the different environmental signals related to pathophysiological conditions. In this regard, an adequate transition from the proinflammatory to the anti-inflammatory response is necessary to counteract retinal neurodegeneration and its subsequent damage that leads to the loss of visual function. Insulin like-growth factor-1 (IGF-1) has been considered as a pleiotropic factor in the retina under health or disease conditions and several effects of IGF-1 in retinal immune modulation have been described. In this review, we provide recent insights of inflammation as a common feature of retinal diseases (AMD, RP and RD) highlighting the role of microglia, exosomes and IGF-1 in this process.

## Role of IGF-1 in retinal inflammation and degeneration

Neuroinflammation is currently considered as an early event in the pathophysiology of many neurodegenerative disorders because despite its essential role in protecting tissues during the early steps of disease, the continuous presence of proinflammatory stimuli induces cellular damage (Glass et al., 2010; Arroba et al., 2016a; Arroba and Valverde, 2017). It is widely accepted that in the central nervous system (CNS) astrocytes and microglia are the cells that play a critical role in neuroinflammation that precedes the neurodegenerative diseases (Cherry et al., 2014; Arroba et al., 2016a). In this scenario, activated microglia and reactive astrocytes participate into the release of different inflammatory mediators including cytokines, chemokines, reactive oxygen species (ROS), and nitric oxide (NO), all of them contributing to the maintenance of a chronic neuroinflammatory milieu that ultimately may be responsible of neurotoxic damage in the CNS (Cuenca et al., 2014).

Insulin-like growth factor-I (IGF-1) is the ligand of the IGF-1 receptor (IGF-1R) which belongs to the tyrosine kinase receptor superfamily and regulates normal developmental growth through endocrine and autocrine/paracrine-mediated mechanisms (Bates et al., 1995). IGF-1 is also a potent survival factor for many tissues (Heemskerk et al., 1999). Particularly, IGF-1 is a neurotrophic peptide in the CNS where it promotes synaptic plasticity, enhances nerve growth and triggers antiapoptotic-mediated signaling cascades (Carro et al., 2003). All these IGF-1 functions are critical for the protection of nerve cells against neurodegenerative processes (Varela-Nieto et al., 2013; Yamamoto et al., 2014). Deficiency in the *IGF1* gene in humans is related with neuronal disorders such as microcephaly, mental retardation, and bilateral sensorineural deafness (Woods et al., 1996; Walenkamp et al., 2005; Netchine et al., 2009).

In several pathologies such as type 1 diabetes mellitus (T1DM) antiinflammatory properties have been attributed to the IGF-1/IGF-IR system in the CNS by counteracting the inflammatory milieu triggered by microglial activation in the hypothalamus (Zhang et al., 2016). However, controversial effects of this peptide have been described regarding its proinflammatory effect in other pathological contexts. In a recent study, IGF-1 was overexpressed in hepatic stellate cells of mice deficient in the *Abcb4* gene, a preclinical model for chronic cholangiopathy. The authors of this work found a higher stimulation of the fibrogenic processes in these double mutant mice which was accompanied by the increased expression of proinflammatory markers together with the presence of infiltrating macrophages in the liver (Sokolovic et al., 2013). Regarding this duality of IGF-1 effects in inflammation, a recent study in zebrafish has evidenced that growth factors including IGF-1 and insulin together with cytokines activate common signaling pathways that are necessary for the reprogramming of Müller glial cells and retinal regeneration upon injury (Wan et al., 2014).

The retina has been considered as a projection of the CNS and, in this tissue, neuroinflammatory processes occur in a similar way as in the brain. In fact, retina and brain share similarities due to their common neuroectodermal origin and derivation from the anterior neural tube and, therefore, both tissues respond similarly to the proinflammatory insults. Based on that, it is conceivable to integrate the retina as a part of the brain (MacCormick et al., 2015).

Retinal degenerative diseases are heterogeneous pathologies; among them, age-related macular degeneration (AMD), retinitis pigmentosa (RP), and diabetic retinopathy (DR) have a high incidence and prevalence in humans (Hernandez et al., 2016; Narayan et al., 2016; Shaw et al., 2017). In fact, a plethora of factors including genetic alterations, aging, vascular defects, chemical insults, oxidative stress, or light-induced damage are responsible of the development of retinal degeneration (Semeraro et al., 2015; van Norren and Vos, 2016). In the pathologies associated to retinal degenerative diseases, progressive degeneration of the retinal neurons, predominantly in photoreceptors, retinal ganglion cells (RGCs), and cells of the retinal pigment epithelium (RPE), results in a severe deterioration of the visual function that in many cases leads to a complete blindness (Nazari et al., 2015).

Inflammatory responses contribute to the pathophysiology of numerous ocular diseases (Dick, 2017). In this regard, RPE cells play a critical role in mediating immune responses to stressing agents such as bacterial endotoxins or proinflammatory cytokines. In fact, persistent inflammation can induce a severe damage in the RPE, thereby contributing to the activation of choroidal neovascularization (CNV), which is observed in more advanced forms of AMD (Chen et al., 2017). In RP, anti-retinal antibodies are associated with the development of cystoid macular edema (Nishikawa et al., 2017) and there are several studies suggesting that the systemic inflammatory profile is altered and may contribute to disease progression (Li et al., 2015; Mori et al., 2017). In addition, recent work supports the involvement of two key factors linking type 2 diabetes mellitus (T2DM) with neurodegeneration; the elevation of proinflammatory cytokines and the onset of insulin/IGF-1 resistance. Moreover, in T2DM, proinflammatory cytokines could be responsible of the disruption in the insulin/IGF-1 signaling pathways in peripheral tissues and the pancreas (Feve and Bastard, 2009). Likewise, accumulation of peripheral proinflammatory mediators, some of which can cross the blood-brain barrier, likely triggers insulin/IGF-1 resistance in the CNS that results in the attenuation of their neuroprotective signaling pathways, thus contributing to the onset of neurodegenerative diseases (Arroba et al., 2011).

Many studies have shown that insulin and IGFs play a relevant role in modulating the balance between growth and survival of the retinal cells (Hernandez-Sanchez et al., 1995; Frade et al., 1996; Alarcon et al., 1998). In the retina, IGF-1 is a potent proangiogenic factor that is present in the neovascular membranes from AMD patients (Lambooij et al., 2003). Moreover, in those patients, elevated concentrations of IGF-1 in plasma have been detected (Machalinska et al., 2011). Likewise, in the majority of experimental models of RP the loss of visual function parallels photoreceptor cell death (Chang et al., 1993; Sancho-Pelluz et al., 2008). In this regard, in the *rd10* experimental mouse model of RP proinsulin delays the death of photoreceptors and prolongs visual function (Corrochano et al., 2008) and, importantly, IGF-1 also decreases apoptosis of photoreceptors in both genetic and experimentally induced RP models (Arroba et al., 2009).

During aging, bioactive IGF-1 circulating levels are reduced, a trend that has been associated with human frailty and cognitive decline (Vestergaard et al., 2014). In the retina, controversies on the beneficial or deleterious effects IGF-1/IGF-1R levels on aging-related degenerative diseases have been reported. Regarding deleterious effects, elderly onset patients with diabetes have lower prevalence of proliferative DR (PDR) than those younger onset patients for similar diabetes duration, which may be related with lower serum IGF-1 levels detected in the older patients (Zhang et al., 2017). Thus, less stringent glycemic control in older onset patients with diabetes may not increase the prevalence of PDR. On the other hand, the evaluation of mice lacking the *Igf1* gene by electroretinography and selective labeling of retinal cells has evidenced an age-related accelerated loss in visual function accompanied by a significant loss of cell contacts between photoreceptors and their postsynaptic cells that may be related to the physiopathology of human IGF-1 deficiency and resistance (Rodriguez-de la Rosa et al., 2012).

## Effects of IGF-1 on microglia in retinal diseases associated to neurodegeneration

The neural retina contains a barrier system, the blood-retinal barrier (BRB), with two components: the inner BRB formed by tight junctions responsible of sealing neighboring capillary endothelial cells, and the outer BRB with tight junctions that restrict the paracellular trafficking between the retinal pigment epithelial cells (Spadoni et al., 2017). Thus, the increase in the permeability of the BRB results in the leak of plasma components into the retina (Eshaq et al., 2017). Importantly, both layers ensure an immune-privileged status of the eye and are also essential for regulating retinal homeostasis and visual function. In this regard, the BRB creates an immunosuppressive milieu that inhibits the activation of immune cells as they cross the barrier and promotes immune privilege (Forrester and Xu, 2012). The outer BRB also expresses immunoregulatory molecules that inhibit lymphocyte activation while the RPE of the BRB secretes immunomodulatory mediators into the aqueous humor that restrain the immune and inflammatory responses within the eye (Sohn et al., 2000). Therefore, under physiological conditions, immune cells of the circulation are not able to enter into the retina to combat with endogenous insults. Instead, the retina has an unique immune defense system consisting of innate immune cells (microglia, perivascular macrophages, and dendritic cells) and the complement system (Ramirez et al., 2017). The function of these cells suggests that they are “gatekeepers” of the retina. Thus, the importance of the research aimed to dissect the function of the different inflammatory processes in the retina and, especially the contribution of microglial-mediated neuroinflammation that antecedes neurodegeneration, could provide useful knowledge for the implement of challenging therapies.

IGF-1 has been associated with the pathogenesis of BRB breakdown. In mice, high intraocular IGF-1 due to its overexpression in the retina increased IGF-1R-mediated signaling resulting in the accumulation of VEGF, up-regulation of vascular intercellular adhesion molecule I and retinal infiltration by bone marrow-derived microglial cells. Altogether these alterations increased vessel paracellular permeability to both low and high molecular weight compounds and correlated with the loss of vascular tight junction integrity. In contrast, mice with chronically elevated serum IGF-1 did not show alterations in the retinal vasculature structure and permeability, indicating that circulating IGF-1 cannot initiate BRB breakdown. Importantly, in human retinas of patients with marked gliosis a strong up-regulation of the IGF-1R was detected, suggesting that therapeutic interventions aimed to counteract local IGF-1 effects may prove successful to prevent BRB disruption (Haurigot et al., 2009).

In 1932 Pio del Rio-Hortega characterized microglial cells, a component of the retinal immune defense system that constitutes approximately 5–12% of the total cells of the CNS, as an exclusive cell type in the brain with phagocytic functions that differs from glial and neuronal cells in morphology (Ginhoux et al., 2013). In the retina, microglial cells are distributed in the inner plexiform and outer plexiform layers (IPL and OPL, respectively), ganglion cell layer (GCL), and nerve fiber layer (NFL), showing highly motile protrusions that survey the surrounding environment (Cheng et al., 2002). Activated microglia has the capacity to move in all directions, but this movement is not accompanied by soma migration. Moreover, under specific circumstances microglia may be translocated to the different retinal layers where the injury is located (Lee et al., 2008; Arroba et al., 2016a; Arroba and Valverde, 2017).

Under physiological conditions, microglial cells are maintained in a resting state characterized by a small cell body and long thin dendrites (Ginhoux et al., 2013) and immunolabelling for Cd-11b. However, microglial cells are chronically activated in retinal diseases such as AMD, DR, and RP (Penfold et al., 2001; Arroba et al., 2011, 2016a) and, as occurs in with the immune cells of the periphery, this activation contributes to boost retinal damage and to accelerate disease progression. Classically, microglial cells coexist in two states, resting and activated (Nayak et al., 2014). The traditional states or definitions of microglia are currently being modified based on new knowledge about their role in pathogenesis. Therefore, microglia can develop a number of different phenotypes and functions aimed to preserve retinal homeostasis in health and disease, mainly depending on their specific environment (Herrera et al., 2015; Du et al., 2017).

Results from *in vitro* experiments have shown that, as occurs with macrophages, activated microglia cells coexist in two distinct phenotypes depending on the stimuli; proinflammatory/classically activated (M1) immunolabelling Iba-1^+^/iNOS^+^ or antiinflammatory/alternatively activated (M2) immunolabelling Iba-1^+^/Arginase-1^+^. M1/proinflammatory microglia releases neurotoxic and/or inflammatory mediators including TNF-α, interleukin-1β (IL-1β), IL-6, and glutamate and increases the expression of inducible nitric oxide synthase (iNOS), all of them exacerbating the death of retinal neurons (Varnum and Ikezu, 2012; Gonzalez et al., 2014). By contrast, M2/immunoregulatory microglia induces retinal repair and regeneration, as well as secretes growth factors and antiinflammatory cytokines aimed to resolve inflammation and ensure the survival of the retinal neurons (Arroba et al., 2011, 2016a,b; Li et al., 2015; Arroba and Valverde, 2017). Under the M2 phenotype, microglia releases the typical antiinflammatory cytokines such as IL-4, IL-13, IL-10, transforming growth factor (TGF)-β and neurotrophic factors such as IGF-1 (Jung and Suh, 2014). To add more complexity to the diversity of microglia polarization stages, several subclasses of M2/immnunoregulatory activation have been identified. The M2a participates in attenuation of inflammation whereas the M2c is involved in restoring or repairing the tissue once the M2a response has effectively completed its participation (Gordon et al., 2003; Chang et al., 2009; Sica and Mantovani, 2012). Finally, a subtype of microglia named M2b is referred to a component involved in the memory immune response which is also able to elicit both pro- and responses (Edwards et al., 2007; Barilli et al., 2014). However, as stated above, this concept initially came from *in vitro* experiments with defined ligands and, therefore, it is difficult to translate to the *in vivo* context where many overlapping phenotypes in inflamed tissues coexist.

As recently reviewed (Labandeira-Garcia et al., 2017), IGF-1 is a mitogenic factor for microglia which also modulates the neuroinflammatory responses from microglial cells by promoting a switch toward the microglial phenotype. Furthermore, the decrease in IGF-1 that occurs with aging has been proposed to contribute to the loss of the capacity of microglia. Therefore, a tight modulation of IGF-1 levels is necessary for the regulation of the neuroinflammatory responses of the microglia since, as stated above, IGF-1 is likely be involved in inflammation in a context-dependent manner (Hotamisligil et al., 1993; Bluthe et al., 2006). Several studies in the retina have found a direct relationship between microglial activation and increased neuronal injury in experimental models of RP, glaucoma, light-induced photoreceptor degeneration, DR, and AMD (Zeng et al., 2005; Glybina et al., 2010). Regarding this issue, recent work of our laboratory and others has provided new insights on the effect of *Igf1* deficiency during aging in the blockade of autophagic flux in the retina which is closely related to neuroinflammation. By using an experimental mouse model of *Igf1* deficiency we demonstrated for the first time that in these mice aging concurs with retinal neuroinflammation. Importantly, in retinas from aged *Igf1*-deficient mice, the inflammatory process concurred with the blockade of the autophagic flux (Arroba et al., 2016c). At the molecular level, we identified elevations in the phosphorylation of mTORC1, as well as in the levels of the autophagy substrate p62, in whole retinal extracts. The corroboration of these results was evidenced by transmission electronic microscopy analysis in which we detected an accumulation of autophagosomes in the INL and OPL layers of the retina in *Igf1*^−/−^ mice at the age of 12 months. Since it was previously reported that in these layers the neural synapsis were disrupted (Rodriguez-de la Rosa et al., 2012), we hypothesized that autophagy might be a necessary process in the neuronal cells of the INL and OPL layers of the retina to preserve photoreceptor connectivity (Shen et al., 2016), a process which is likely negatively affected by the proinflammatory milieu. Remarkably, in these layers the accumulation of autophagosomes concurred with the presence of activated amoeboid microglia (Iba-1^+^) (Arroba et al., 2016a). As stated above, these are the critical layers where the cells responsible for the amplification of the synaptic transmission, which are highly sensitive to the inflammatory environment, are located (Noailles et al., 2016). Altogether, the results of our work in *Igf1*-deficient mouse model have identified for the first time autophagy as an adaptive response against the chronic activation of the inflammasome in microglial cells of the retina during aging. In this regard, we and others have proposed that autophagy can be considered as a relevant defense against deterioration of the retinal synapses and visual function (Noailles et al., 2014; Arroba et al., 2016c). In this concept, in *Igf1* deficient mice, the malfunction of microglia during aging can lead to the establishment of a chronic low-grade inflammatory environment, favoring the onset and further progression of retinal degeneration.

### Aged-related macular edema

AMD is the progressive damage in the macular region of the retina. Although the exact mechanisms involved in its pathogenesis have not been completely elucidated, chronic inflammation together with oxidative stress play a major role (Kauppinen et al., 2016). The infiltration of macrophages from the peripheral circulation is a unique and interesting component of the inflammatory component of AMD (Funk et al., 2009; Lavalette et al., 2011). In this regard, one of the characteristics of atrophic or “dry” AMD is the accumulation of microglia/macrophages in the outer retina and subretinal space (Penfold et al., 1987, 2001). Previous investigations have reported that eyes of patients with AMD have elevated concentrations of proinflammatory cytokines that modify the activity of CNV (Hageman et al., 2001). Histological studies have revealed that chronic inflammation occurs at the retinal pigment epithelial/choroidal interface in eyes with early signs of AMD such as drusen (Hernandez-Zimbron et al., 2018). Furthermore, in exudative AMD a higher up regulation of inflammatory cytokines/chemokines from RPE cells and macrophages/monocytes positively and negatively control CNV activity (Kauppinen et al., 2016). Regarding the involvement of the IGF-1 system in AMD, a case-control study involving 962 subjects showed that in non-diabetic individuals from the Age-Related Eye Disease Study (AREDS) Genetic Repository the SNP rs2872060 in the IGF-1R was significantly associated with the risk for advanced AMD and this association remained significant after patient stratification by the two types of the disease: neovascularization and geographic atrophy (Chiu et al., 2011). Interestingly, the risk allele (G) showed an additive effect and a significant interaction with BMI on the risk for neovascularization, but not for geographic atrophy. On the other hand, another study has reported increased levels of insulin-like growth factor binding protein 2 (IGFBP-2) and IGF-1 in exudative AMD eyes, indicating that defects in the expression of IGF-related molecules may be involved in the disease pathogenesis for exudative AMD (Cha et al., 2013).

### Retinitis pigmentosa

RP belongs to a retinal degenerative group of inherited diseases that affects about 2.5 million people worldwide and is characterized by a progressive photoreceptor cell death that ultimately leads to a severe vision loss (Dias et al., 2018). Initially, the cell death affects only to photoreceptor cells; however, during the progression of the disease abnormalities in the RPE and the cones are detected (Campochiaro and Mir, 2018). In addition to mutations in 70 different genes, a proinflammatory component is also a hallmark of the pathogenesis of RP [http://www.retnet.org (latest entry 2017)] (Gupta et al., 2003; Whitcup et al., 2013; Yoshida et al., 2013; Eandi et al., 2016). In fact, the levels of proinflammatory cytokines have been found markedly elevated in both the vitreous and aqueous humor from RP patients (Yoshida et al., 2013), supporting the interaction between photoreceptor cell death and intraocular inflammation. In this context, hyperactivation of microglial cells has been demonstrated to play an important role in the photoreceptor neurodegeneration in animal models of RP (Peng et al., 2014). A recent study using on live-cell imaging in the *rd10* mouse model of RP has identified that during the early stages of the disease microglia is able to migrate, interact with, and phagocyte non-apoptotic photoreceptors, after which it becomes hyperactivated and promotes the loss of non- and apoptotic photoreceptors (Zhao et al., 2015). The authors of this study propose that primary microglial phagocytosis could be a potential cellular target for therapy. Remarkably, microglial activation mediates photoreceptor loss not only in RP, but also in AMD and DR in both preclinical animal models as well as in human patients (Zhao et al., 2015). In this regard, the emerging therapeutic strategies to combat degenerative diseases of the retina are focused in the attenuation of microglial activation (Karlstetter et al., 2015). However, to achieve this purpose more research is needed to decipher new molecular mechanisms involved in microglial activation during retinal degenerative diseases.

The effect of IGF-1 as a neuroprotective factor has been also demonstrated during RP progression. In this regard, our previous work has found a beneficial effect of IGF-1 since it attenuated reactive gliosis and apoptosis in *ex vivo* retinal explants from *rd10* mice (Arroba et al., 2011). Importantly, the elimination of retinal microglia by using of clodronate-filled liposomes reduced the efficacy of IGF-1 on photoreceptor viability. Likewise, IGF-1 was not able to inhibit the reactive Müller gliosis in absence of microglial cells. Altogether, these results indicate that the beneficial effects of IGF-1 are mediated, at least in part, by the microglia (Arroba et al., 2011, 2014). These findings suggest first a critical role of the crosstalk between microglia and Müller glial cells during neuroprotection (Arroba et al., 2014) and, second, that microglia is necessary for the neuroprotective effects of IGF-1 in the dystrophic retina (Arroba et al., 2011).

### Diabetic retinopathy

Among the microvascular complications of diabetes, DR is the most undesirable one. DR is a progressive retinal disease and a leading cause of blindness in diabetic patients due to degeneration of both the retinal vasculature and retinal neurons (Hernandez et al., 2016). DR is broadly classified into two stages: non-proliferative DR (NPDR) and proliferative DR (PDR). This classification is determined by the presence of neovascularization in the retina (Nentwich and Ulbig, 2015). NPDR typically precedes PDR and is divided into the following stages: mild, moderate, severe, and very severe based on the probability of disease progression to PDR. PDR is defined by the presence of neovascularization and is divided into the following stages: early, high risk, and severe neovascularization. Growing evidence suggests that neuroinflammation plays an early role in mediating neuronal and vascular pathology in DR (Oellers and Mahmoud, 2016).

During DR progression, the retina is affected by both external signals such as high glucose, advanced glycation-end products (AGEs) and circulating proinflammatoty cytokines (Dong et al., 2014), as well as by intrinsic signals (Sappington et al., 2006; Legacy et al., 2013). In the context of neuroinflammation, high levels of proinflammatory cytokines are detected in the retina in different animal models of DR (Li and Puro, 2002) and in retinas from diabetic patients (Arroba et al., 2016a; Hernandez et al., 2016). We have recently reported that during DR progression in diabetic *db/db* mice there is a switch from antiinflammatory to proinflammatory polarized microglia in parallel to the deterioration of visual function (Arroba et al., 2016a). In addition, in this mouse model, retinal gliosis was also detected suggesting that in addition to microglia, macroglia likely contributes to this switch boosting inflammation and promoting reactive gliosis and this scenario concurs with the death of retinal cells by apoptosis (Bogdanov et al., 2014). Whether IGF-1 is able to modulate the dynamics of microglia polarization in the setting of DR is still unknown. This is a controversial issue due to the opposite effects of IGF-1 reported in the retina with deleterious effects on the retinal vasculature (Hellstrom et al., 2002), but positive effects in the survival of retinal neurons (Kermer et al., 2000). Villacampa and co-workers have generated a transgenic mice overexpressing *Igf1* in photoreceptors to evaluate the deleterious effects of the persistent elevation of intraocular IGF-1 on retinal functionality (Villacampa et al., 2013). They showed a progressive decline in the electroretinogram amplitudes in *Igf1* transgenic animals, leading to a complete loss of response with aging. Importantly, markers of retinal stress, gliosis, and microgliosis were already present at early stages of the disease before the detection of major vascular alterations. Despite these interesting findings, the translation of this study to the human retinal diseases deserves further research.

## IGF-1 and intercellular communications in retinal diseases associated to neuroinflammation: involvement of extracellular vesicles

Extracellular vesicles (EVs) have been related to some pathologies since they are involved in a variety of immune activities having protective or detrimental properties. It is also well known that EVs have a heterogeneous molecular composition including DNA, mRNAs, micro RNAs (miRNAs), integrins, cytokines, bioactive lipids, and organelles, being these molecules similar to those of their parental cells (Colombo et al., 2014). EVs can be detected in biological fluids such as plasma (Sharma et al., 2018) and the cerebral spinal fluid (McKeever et al., 2018). Of relevance, microglial EVs retain many features of the original cells where they come from and, consequently, they are considered as a “liquid biopsy” that provide relevant information about the status of activation of their microglial parental cells during the course of the neurodegenerative processes (Kalani et al., 2014; Nigro et al., 2016). Furthermore, the EVs cargo is modulated by the pathophysiological environment in a way that they become vehicles loaded with pathogenic cargo such as aggregating proteins in neurodegenerative diseases (Schneider and Simons, 2013), oncoproteins in cancer (Nakano et al., 2015), or inflammatory cytokines in neuroinflammatory diseases (Verderio et al., 2012; Prada C. E. et al., 2013).

EVs present a small size (< 1,000 nm for microvesices and < 100 nm for exosomes) which favors the migration from the site of discharge and allows the communication between distant cells (Mulcahy et al., 2014). In the brain, EVs released from the surface of reactive microglia provoke glial activation due to the induction of an inflammatory reaction in target glial cells, both microglia and astrocytes in an autocrine and paracrine manner, respectively (Antonucci et al., 2012). The inflammatory reaction induced by microglial cells-derived EVs is related to their ability of transferring mRNAs encoding inflammatory cytokines such as IL-1β (Prada I. et al., 2013). However, little or no uptake of EVs or exosomes occurs in astrocytes, and there are no evidences for microglia to astrocyte transfer of nucleic acids through EVs. It should be noted that EVs production is not exclusively restricted to microglial cells because, as it will be detailed below, the RPE is proactive secreting EVs.

Recent investigations have described that EVs from the aqueous humor of patients with AMD present specific proteins suggesting that EVs could be used as predictor biomarkers of this retinal disease (Biasutto et al., 2013; Kang et al., 2014; Tong et al., 2016). In another study, the molecular processes associated to aged RPE have revealed increased in autophagy and exocytotic activity that was associated with the presence of autophagic EVs markers (Atienzar-Aroca et al., 2016; Kannan et al., 2016). In this regard, it has been demonstrated that RPE cells release EVs under oxidative stress environment and this is accompanied with a high expression of VEGFR in their membrane and increased VEGFR mRNA cargo (Wang et al., 2009). Moreover, EVs can transport proteins related with essential signaling pathways such as mitogen-activated protein kinase (MAPK), nuclear factor kB (NFKB), and protein kinase B (AKT), as well as miRNAs (i.e., miR-294 or miR-302) into retinal microvascular endothelial cells (Tong et al., 2016). These miRNAs and proteins play important roles in processes associated with cell proliferation and, taking this into account, it has been suggested that RPE cells-derived EVs could contribute to the development of CVN (Tong et al., 2016). Beside the active role of RPE cells in exosomes secretion, the Hajrasouliha's study has revealed that exosomes from retinal astrocytes from non-pathologic mouse contain several antiangiogenic factors (PEDF and endostatin) that block the development of CNV in a laser-induced mouse model by targeting both macrophages and vascular endothelial cells (Hajrasouliha et al., 2013). Altogether, these results strongly suggest that, in the eye, exosomes derived from different ocular cells may play an important role for modulating the balance of anti- and pro-angiogenesis and the integrity of vision function.

EVs have also been involved in photoreceptor cells of the vertebrate retina which are continuously renewed by the addition of membranes at the base of the outer segments (OS) and removal of the older discs form the distal end (Besharse et al., 1977). Studies in retinas of other species such as *Xenopus* have shown that OS-bound proteins are continuously sorted and trafficked from the endoplasmic reticulum and trans-Golgi network as cargo in EVs from the inner segment toward the OS (Papermaster et al., 1985). The existence of interactions between EVs cargo proteins and proteins involved in the transport machinery is supported by several studies in animal models demonstrating that a defect in only one of these proteins can lead to a total loss of polarity in the protein trafficking resulting in photoreceptor death (Hagstrom et al., 1999; Deretic, 2006).

Actually, there are no studies in RP retinal degeneration disease linked to EVs secretion. Only a recent study which analyzes RP in combination with hearing loss describes a novel syndrome caused by biallelic mutations in the “exosome component 2” (*EXOSC2*) gene (Giunta et al., 2016). This study was performed in three patients from two German families without any relationship that were affected by a Mendelian disorder characterized by a progressive sensorineural hearing loss, childhood myopia, early onset RP, short stature, hypothyroidism, premature aging, recognizable facial gestalt, and mild intellectual disability. The exome sequencing identified homozygous or heterozygous missense variants in the *EXOSC2* gene in all three patients. *EXOSC2* encodes the “ribosomal RNA-processing protein 4” (RRP4), one of the core components of the RNA exosome. The phenotype associated to EXOSC2 was characterized by only a minimal overlap with previously reported diseases associated with mutations in the RNA exosome core component genes *EXOSC3* and *EXOSC8* (Giunta et al., 2016). The clinical consequences of altered RNA exosome function during RP is a novel condition which deserves further studies.

Although the role of EVs in the IGF-1 system in the retina is still unknown, it has been recently reported that mesenchymal stem cells-derived exosomes activate several signaling pathways which are involved in wound healing (AKT, MAPK, and STAT3) and are able to induce the expression of IGF-1 among other growth factors (Shabbir et al., 2015). In another study, exosomes derived from cardiomyocytes of a type 2 diabetic rat GK (GK-exosomes) inhibited mouse cardiac endothelial cells proliferation, migration and tube-like formation, whereas all these parameters were promoted by exosomes from non-diabetic rats (WT-exosomes) (Wang et al., 2014). Mechanistically, GK-exosomes encapsulated higher levels of miR-320 which functionally down-regulated target genes in mouse cardiac endothelial cells, one of which was IGF-1. Altogether these results conclude that in an experimental model of T2DM cardiomyocytes have an antiangiogenic function which is mediated by IGF-1 and involves the participation of exosomes in the transference of the miR-320 into endothelial cells. More research will be necessary to unravel cellular interactions via EVs within the retina and the processes modulated by IGF-1.

## Emerging insights on the role of IGF-1 in targeting inflammation in retinal diseases

It has been reported that in the brain the antiinflammatory effects of some compounds rely in their capability to guide the polarization of microglia toward an antiinflammatory phenotype (Chio et al., 2015). In animal models of several diseases associated to inflammation, including neurodegenerative diseases, it has been described how microglia changes the polarization state under treatment with omega-3 polyunsaturated fatty acids by decreasing the production of neurotoxic and proinflammatory molecules (Calviello et al., 2013; Serini and Calviello, 2016). Based on that, activation of microglial cells toward the antiinflammatory response and the subsequent reduction of proinflammatory cytokines are promising approaches for retinal neuroprotection in several models of retinal degeneration such as AMD, DR, and RP. In fact, several compounds from distinct origins elicit protective effects against inflammation, ischemia, light-, oxygen-, and age-associated pathologies of the neural retina in animal models (SanGiovanni and Chew, 2005; Tuo et al., 2009; Dornstauder et al., 2012) by their ability to resolve inflammation; all this effects being essential to avoid the progression of these retinal diseases. Not only the anti-inflammatory microglia is directly responsible of the protection against neuroinflammation since it has been described that microglia can elicit indirect effects. In this regard and, as stated above, we have demonstrated the requirement of the presence of microglia for the neuroprotective effect of IGF-1 in a mouse model of RP (Arroba et al., 2011).

AMD progression concurs with chronic and pathophysiological low-grade inflammation associated with a high cellular metabolism which contributes to generate ROS, oxidized lipoproteins, advanced glycation end-products, and apoptotic cells (Xu et al., 2009). Interestingly, in early AMD, microglia acts mainly with scavenger and antiinflammatory properties. However, in late AMD in diabetic rats, microglia promotes the angiogenic activity by increasing the expression of VEGF among different growth factors as well as ROS (Ma et al., 2007).

Recently, Cotter's laboratory and ours have reported that different kind of compounds (progesterone, sp^2^-iminosugar dodecylsulfoxide or chemical inhibitors of protein tyrosine phosphatase 1B) are able to act on microglial cells to reduce the proinflammatory milieu by decreasing TNF-α, IL-1β, and iNOS levels and stimulate the antiinflammatory phenotypes by increasing CD206/MRC1 and arginase-1 in mouse models of models of RP (*rd10*) or DR (*db/db*), respectively (Arroba et al., 2016a; Roche et al., 2016). Regarding DR, the treatment of retinal explants from *db/db* mice with the sp^2^-iminosugar derivative compound R-DS-ONJ ameliorated the reactive gliosis already detected in those retinas, as well as increased the antiinflammatory marker arginase-1, thereby reflecting a regression toward an early stage of DR (Arroba et al., 2016a). In this study, we also achieved the mechanism of action of the R-DS-ONJ compound by performing *in vitro* cell-based approaches aimed to mimic the proinflammatory environment associated to DR. By using Bv.2 mouse microglia cells stimulated with LPS, a proinflammatory stimulus that resembles the *in vivo* situation in *db/db* mice in the course of DR, we found that the co-treatment of LPS with IL4/IL3 (M2 cytokines) or the sp^2^-iminosugar dodecylsulfoxide *R*-DS-ONJ ameliorated the microglial proinflammatory phenotype induced by endotoxemia, as reflected by marked decreases in the levels of nitrites, iNOS mRNA and protein expression as well as by reductions in mRNAs encoding proinflammatory cytokines. At the molecular level, a cocktail of antiinflammatory cytokines or *R*-DS-ONJ reduced LPS-mediated activation of stress kinases (JNK and p38 MAPK) and prevented the degradation of IkBα and the nuclear translocation of the proinflammatory transcription factor NFκB. These two studies have provided new insights on targeting neuroinflammation in the retina by potentiating the polarization state of microglia that might be a promising therapeutic strategy to delay and/or prevent the deterioration of visual function in patients. This interesting issue deserves further research.

Although a direct role of IGF-1 as a potential therapy for targeting the polarization of microglia in the retina has not been reported, there are indirect evidences of its involvement in the dynamics of microglia polarization stages. For instance, mitochondrial toxins inhibited part of the IL-4-induced alternative activation in primary cultures of mouse microglia including the induction of arginase-1 and IGF-1 and, therefore, the counteraction of the LPS induced cytokine release was abolished (Ferger et al., 2010). Also, it is well known that IGF-1 actions are slowed during aging (Lee et al., 2013) and, as we have mentioned above, under this condition the deficiency of *Igf1* in mice reflects an unbalanced anti-inflammatory vs. proinflammatory response in the retina that parallels the retinal degeneration (Arroba et al., 2016a). In addition, new concepts on the modulation of microglial polarization by mitochondrial metabolism have emerged (Orihuela et al., 2016) and in this regard IGF-1-mediated metabolic actions may also play a key role. Future research in the field of neuroinflammation will provide new insights on this important issue.

## Concluding remarks

**Figure 1:**
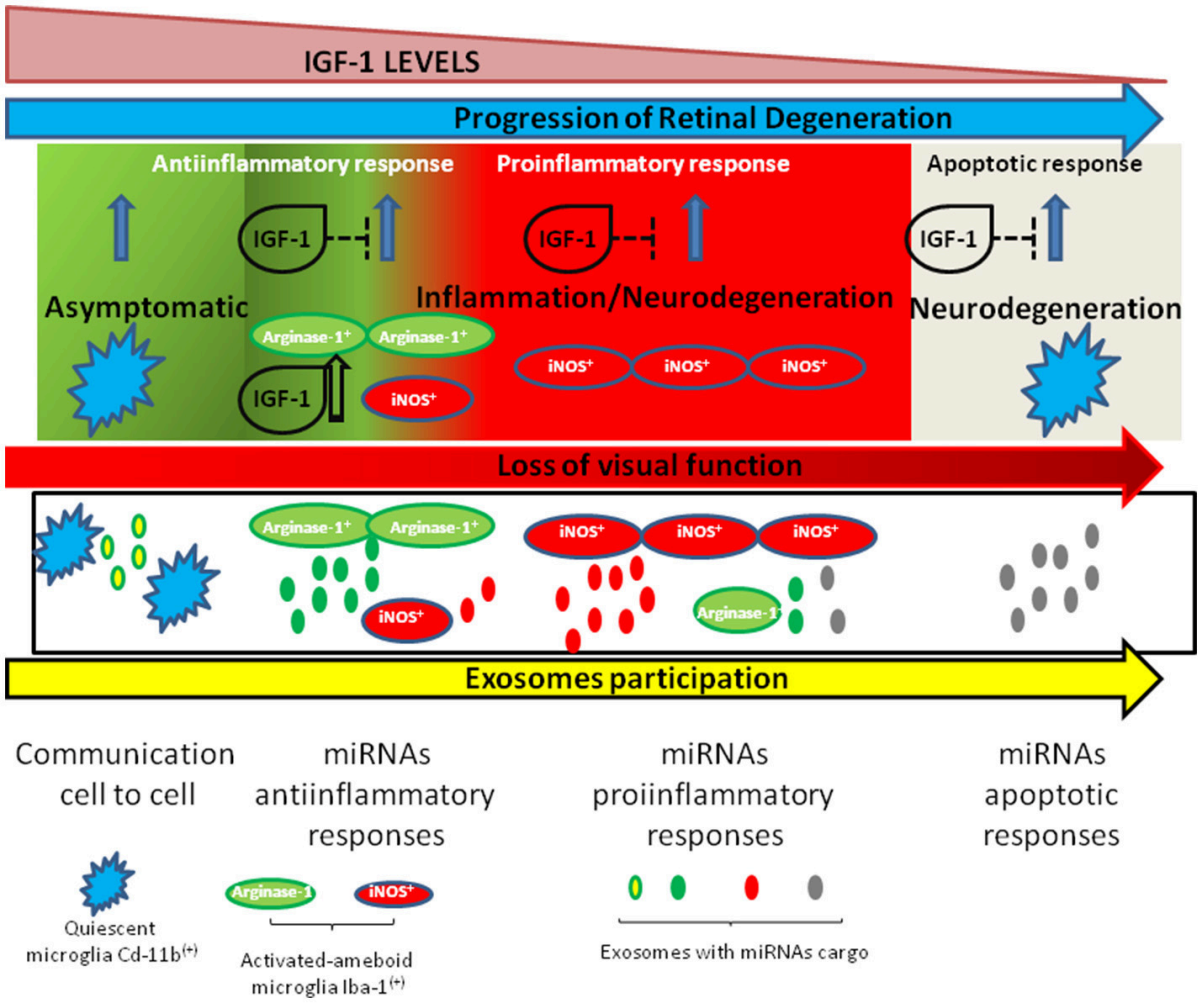
Schematic representation of changes in microglia during retinal neurodegeneration and the role of IGF-1 in this process. IGF-1 levels decrease along aging and in some neurodegenerative diseases and this is associated to a switch in microglia polarization toward a proinflammatory status. In this context, exosomes participate into cell-to-cell communications as vehicles which transport anti- or proinflammatory miRNAS.

The progression of retinal degeneration is mediated by the dynamics of microglia polarization in the early steps of almost all retinal diseases. In this context, and as schematized in Figure 1, in the retina the immune responses must be tightly controlled since they are responsible for a better or worse prognostic of diseases such as DME, RP, or DR. Thus, targeting neuroinflammation through the IGF-1/IGF-1R system and/or additional pharmacological strategies might conduct the regression of retinal degeneration and represents a challenging therapeutic strategy.

## Author contributions

AIA researched data and wrote and reviewed the manuscript. ÁMV provided funding, wrote and reviewed the manuscript. AC-C and MA-D reviewed the manuscript. ÁMV and AIA are responsible for the integrity of the work as a whole.

### Conflict of interest statement

The authors declare that the research was conducted in the absence of any commercial or financial relationships that could be construed as a potential conflict of interest.
